# Giant hepatic hemangioma in a Japanese adult patient reduced by sirolimus therapy

**DOI:** 10.1007/s12328-025-02183-2

**Published:** 2025-08-01

**Authors:** Mio Tsuruoka, Masashi Ninomiya, Jun Inoue, Kosuke Sato, Keishi Ouchi, Kengo Watanabe, Kotaro Doi, Tomoya Sasazaki, Atsushi Masamune

**Affiliations:** Division of Gastroenterology, Tohoku University Graduate School of Medicine, 1-1 Seiryo-machi, Aoba-ku, Sendai, 980-8574 Japan

**Keywords:** Giant hepatic hemangioma, Sirolimus, mTOR inhibitor

## Abstract

Although observation is the standard management strategy for hepatic hemangiomas (HHs), surgical intervention may be indicated in cases of progressive enlargement, lesions > 50 mm, those causing compressive symptoms, or those associated with Kasabach–Merritt syndrome. Here, we report the case of a 51-year-old woman with a giant HH occupying almost the entire liver with a volume of 6572.5 mL. The patient presented with severe compressive symptoms and coagulopathy; however, surgical resection was infeasible. Because she did not develop liver failure, transplantation was not indicated, and no effective treatment options were available. Sirolimus, a mammalian target of rapamycin inhibitor, has been used to treat intractable lymphatic disorders and congenital vascular malformations; however, there have been no previous reports on its use in adult giant HHs. Moreover, sirolimus was administered, which resulted in remarkable tumor shrinkage. This suggests that sirolimus may be a valuable therapeutic option for adult patients with giant HHs in whom conventional treatments are not applicable.

## Introduction

Hepatic hemangioma (HH) is the most common benign liver tumor, with a reported prevalence ranging from 0.4 to 20.0% [1–3]. Usually, it is incidentally discovered during routine health check-ups and remains asymptomatic, requiring no specific treatment [4–6]. However, in lesions > 50 mm in diameter, symptoms, such as abdominal pain and nausea, may occur because of compression of adjacent organs, intratumoral hemorrhage, thrombosis, or tumor rupture [7, 8]. Tumors > 100 mm have been reported to present a higher risk of symptomatic manifestations [9]. In rare instances, extensive thrombus formation within a hemangioma can lead to the abnormal consumption of platelets and coagulation factors, resulting in disseminated intravascular coagulation (DIC), known as Kasabach–Merritt syndrome [10–12]. Surgical resection is indicated in patients presenting with symptoms, severe complications, or evident tumor growth [13–16]. Currently, there are no established nonsurgical treatment options for such patients. Sirolimus, also known as rapamycin, is a specific inhibitor of the mammalian target of rapamycin (mTOR), a serine/threonine kinase that regulates angiogenesis, cell growth, and cell proliferation [17, 18]. In Japan, sirolimus was approved in January 2024 for the treatment of intractable vascular tumors and malformations. This approval was based on the results of a large-scale multicenter prospective phase III clinical trial that evaluated the efficacy and safety of sirolimus over 2 years in pediatric and adult patients with symptomatic low-flow vascular malformations [19]. Therefore, venous malformations are recognized as an indication for sirolimus therapy. HHs are predominantly cavernous hemangiomas classified as venous malformations, according to the International Society for the Study of Vascular Anomalies (ISSVA) classification system [20, 21]. Although there have been several reports on the use of sirolimus in combination with other agents for the treatment of infantile giant HHs, there are no reports of sirolimus monotherapy being used to treat HHs in adults [22–26]. Here, we report the rare and valuable case of a 51-year-old woman with abdominal pain due to a giant HH measuring 356.0 mm in diameter, with a total tumor volume of 6572.5 mL. Surgical resection was infeasible and the patient was not a candidate for liver transplantation. Sirolimus monotherapy was initiated, resulting in an approximately 35% reduction in tumor volume after 30 weeks. Knowingly, this is the first report demonstrating the potential use of sirolimus as a pharmacological treatment for unresectable giant HHs in adults.

## Case report

**Fig. 1:**
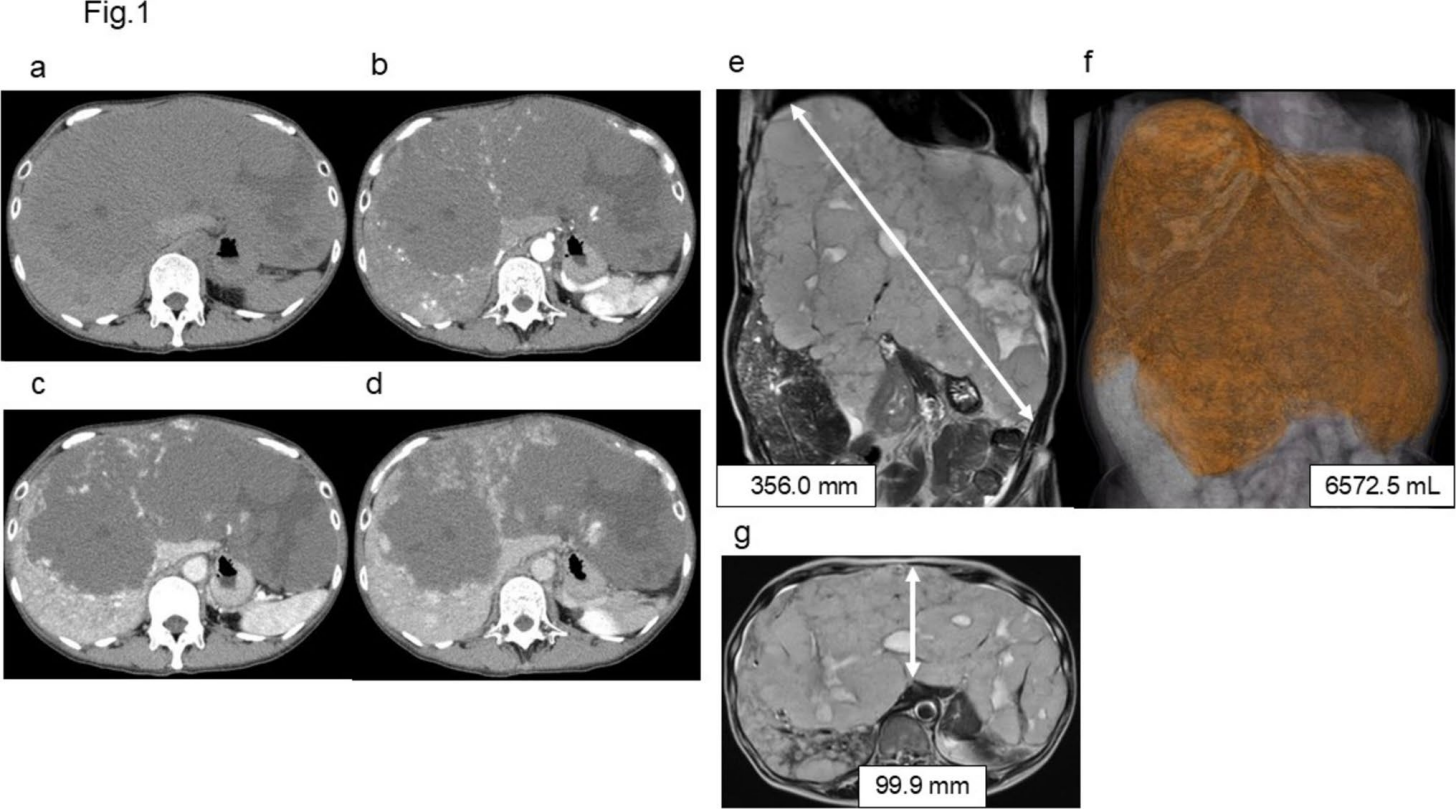
Initial contrast-enhanced CT of the giant hemangioma obtained at our hospital (**a–d**). **a** Plain image. **b** Hepatic arterial phase. **c** Portal venous phase. **d** Delayed phase. T2-weighted MRI shows the giant hemangioma immediately before initiation of sirolimus treatment (**e–g**). **e** The maximum diameter is 356.0 mm. **f** The volume is 6572.5 mL. **g** The distance to the anterior abdominal midline is 99.9 mm

The patient was a 51-year-old woman. She was a former nurse and had undergone a cesarean section at the age of 39. Her family history included lung cancer in her father, liver cancer in her paternal grandmother, colorectal cancer in her maternal uncle and aunt, and cervical cancer in her sister. There was no family history indicative of hereditary or systemic disorders, including Osler–Weber–Rendu syndrome. Clinical examination revealed no evidence of bleeding tendencies involving the skin, mucous membranes, or other organs. She had no history of regular medication use. In 2008, she developed epigastric pain and underwent evaluation at another hospital, leading to a diagnosis of HH with a maximum diameter of 150.0 mm. A gradual increase in size was observed beginning in 2012. However, the patient temporarily discontinued follow-up due to childbirth. In 2017, she resumed outpatient visits with postprandial abdominal pain as the chief complaint. The hemangioma continued to show a progressive increase in size thereafter. After relocation, the patient was referred to our hospital in 2018. At that time, the maximum diameter of the hemangioma had increased to 330.0 mm and coagulation abnormalities were observed. Specifically, the patient’s prothrombin time-international normalized ratio (PT-INR) was 1.15–1.25, fibrinogen (FBG) was 100–120 mg/dL, fibrinogen/fibrin degradation products (FDP) was 30–60 μg/mL, D-dimer was 10–20 μg/mL, and platelet count was 140–190 × 10^3^/μL. These coagulation abnormalities persisted, and the hemangioma continued to grow. Surgical resection was deemed infeasible because of the minimal residual volume of the normal liver tissue. The initial contrast-enhanced computed tomography (CT) images obtained at our hospital are shown in Fig. 1a–d. From the early phase to the delayed phase, the contrast enhancement increased from the periphery toward the center of the lesion, which was a typical finding of HH. Furthermore, giant HHs may show internal degeneration, resulting in a heterogeneous appearance on T2-weighted MRI images, as in this case [27].

Shortly after initiating follow-up at our institution, we administered propranolol, a therapeutic option for infantile HHs, for 1 year and 8 months; however, no therapeutic effect was observed. In the event of hepatic failure, we decided to continue monitoring the patient for liver transplantation. Although the abdominal pressure symptoms worsened, the clinical data did not meet the criteria for insurance-covered liver transplantation in Japan. Therefore, if liver transplantation was to be pursued, it would be conducted as a living-donor transplant under self-funded treatment.

In January 2024, sirolimus received insurance approval in Japan for the treatment of intractable vascular tumors and malformations. Sirolimus was initially approved for lymphangioleiomyomatosis treatment. Since HHs are typically cavernous hemangiomas, they are considered venous malformations by the classification. After discussing with the patient and her family, we decided to initiate sirolimus treatment. Pretreatment magnetic resonance imaging (MRI) of the giant HH is shown in Fig. 1e–g. The lesion had enlarged further, with a maximum diameter of 356.0 mm and volume of 6572.5 mL. The tumor volume was measured using Ziostation2 (Ziosoft Inc., Tokyo, Japan). Additionally, multiple hemangiomas were observed in the surrounding normal liver parenchyma.

**Fig. 2:**
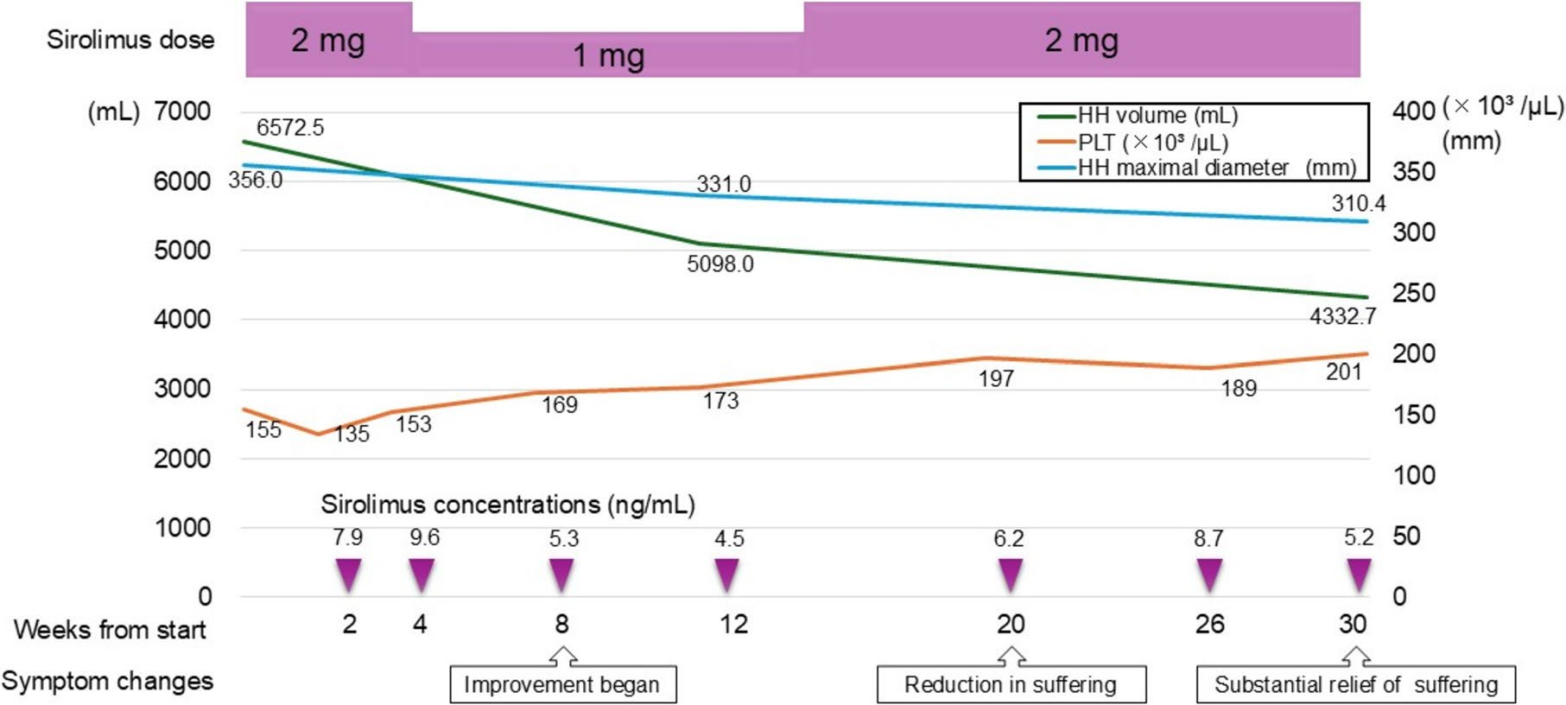
Clinical course. HH, hepatic hemangioma; PLT, platelet

Sirolimus was administered at 2 mg, as per the recommended dose for adult venous malformations. The laboratory findings prior to the initiation of treatment are presented in Table 1. At that time, her Child–Pugh score was 5, corresponding to class A. On day 11 after initiation, oral mucositis appeared. This condition was manageable with dexamethasone oral ointment and did not significantly impair the quality of life. As sirolimus blood levels stabilized approximately 10 days after initiation, the first blood level was measured 2 weeks after starting treatment (7.9 ng/mL). Since the level was within the therapeutic target range of 5.0–15.0 ng/mL, administration of sirolimus was continued. At 4 weeks, the blood level was 9.6 ng/mL, but the patient’s neutrophil count had decreased from a baseline of 1.10–2.0 × 10^3^/μL to 0.85 × 10^3^/μL. Therefore, the sirolimus dose was reduced to 1 mg. After 2 weeks, the neutrophil count returned to baseline. At 8 and 12 weeks, the sirolimus level was 5.3 ng/mL and 4.5 ng/mL, respectively, indicating a downward trend. However, as the neutrophil count remained stable without further decline, the dosage was increased to 2 mg at 14 weeks. After 26 weeks, the coagulation parameters showed marked improvement. PT-INR was 1.01, FBG was 282 mg/dL, FDP was 14.4 μg/mL, D-dimer was 9.2 μg/mL, and platelet count was 189 × 10^3^ /μL. The clinical course over the first 30 weeks is summarized in Fig. 2.

**Table 1:** Laboratory findings before sirolimus treatment

	Patient’s data	Normal range
WBC (× 10^3^/μL)	2.2	3.3–8.6
Neut (× 10^3^/μL)	1.11	1.62–6.54
RBC (× 10⁶/μL)	3.95	3.86–4.92
Hb (g/dL)	11.9	11.6–14.8
PLT (× 10^3^/μL)	155	158–348
T-BIL (mg/dL)	1.2	0.4–1.5
D-BIL (mg/dL)	0.1	≤ 0.2
ALP (U/L)	89	38–113
γ-GTP (U/L)	85	9–32
AST (U/L)	26	13–30
ALT (U/L)	17	7–23
LDH (U/L)	149	124–222
BUN (mg/dL)	16	8–20
CRE (mg/dL)	0.84	0.46–0.79
TP (g/dL)	6.8	6.6–8.1
ALB (g/dL)	4.4	4.1–5.1
Na (mmol/L)	141	138–145
K (mmol/L)	4.3	3.6–4.8
Cl (mmol/L)	105	101–108
PT (%)	78.8	70.0–130.0
PT-INR	1.14	0.80–1.20
APTT (sec)	33.5	24.3–34.6
FBG (mg/dL)	148	200–400
FDP (μg/mL)	43.2	≤ 5.0
D-dimer (μg/mL)	20.0	≤ 1.0
CRP (mg/dL)	0.06	≤ 0.14

*WBC* white blood cell, *Neut* neutrophil, *RBC* red blood cell, *Hb* hemoglobin, *PLT* platelet, *T-BIL* total bilirubin, *D-BIL* direct bilirubin, *ALP* alkaline phosphatase, *γ-GTP* gamma transpeptidase, *AST* asparate aminotransferase, *ALT* alanine aminotransferase, *LDH* lactate dehydrogenase, *BUN* blood urea nitrogen, *CRE* creatinine, *TP* total protein, *ALB* albumin, *Na* sodium, *K* potassium, *Cl* chlorine, *PT* prothrombin time, *PT-INR* prothrombin time-international normalized ratio, *APTT* activated partial thromboplastin time, *FBG* fibrinogen, *FDP* Fibrinogen/fibrin degradation products, *CRP* c-reactive protein

**Fig. 3:**
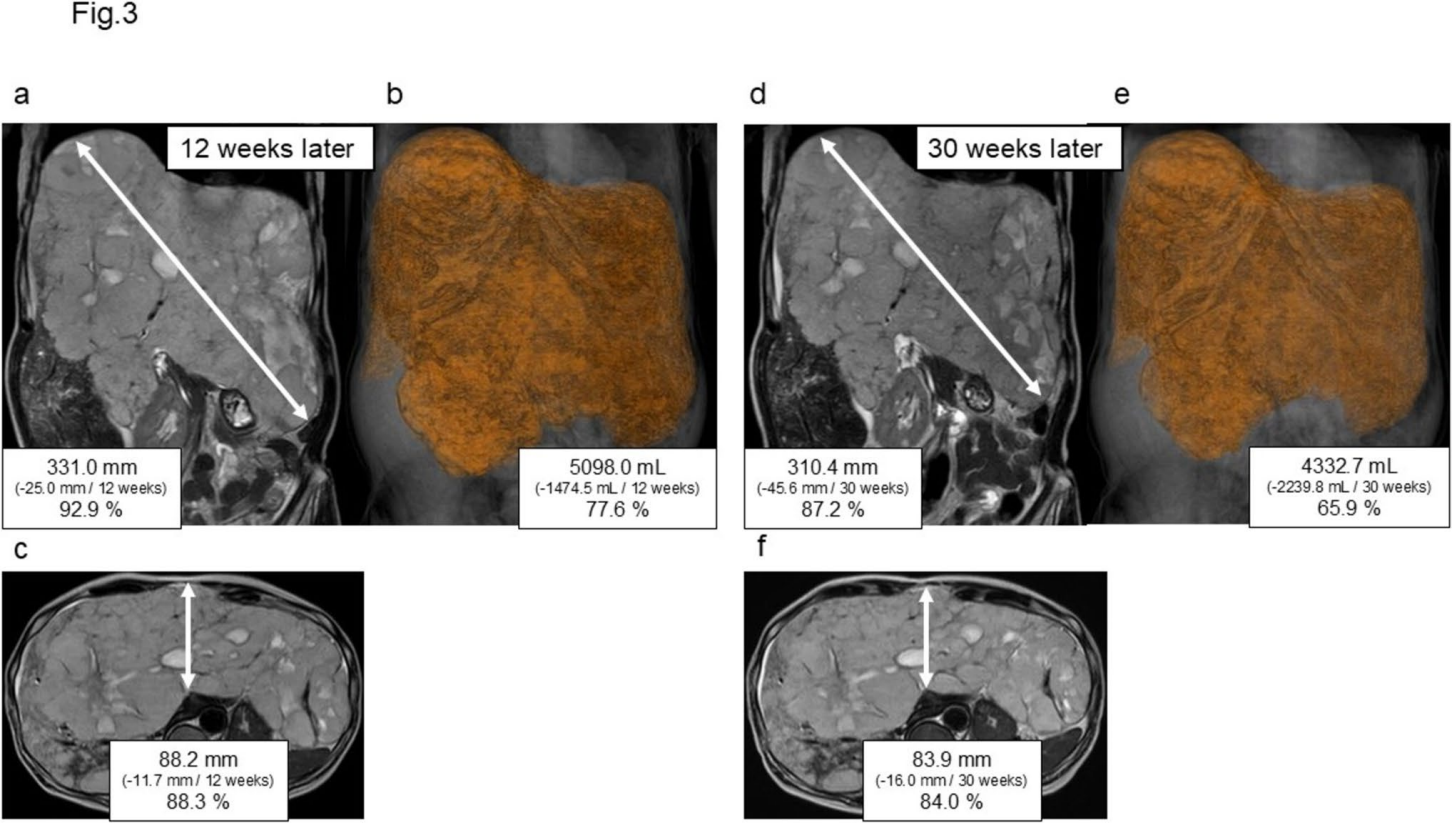
MRI of the giant hemangioma obtained at 12 (**a–c**) and 30 (**d–f**) weeks after initiation of sirolimus therapy. **a** The maximum diameter is 331.0 mm, representing a decrease of 25.0 mm from baseline. **b** The volume decreased to 5098.0 mL, a reduction of 1474.5 mL from baseline, corresponding to 77.6% of the initial volume. **c** The distance to the anterior abdominal midline is 88.2 mm. **d** The maximum diameter is 310.4 mm, representing a decrease of 45.6 mm from baseline. **e** The volume decreased to 4332.7 mL, a reduction of 2239.8 mL from baseline, corresponding to 65.9% of the initial volume. **f** The distance to the anterior abdominal midline is 83.9 mm, representing a decrease of 16.0 mm from baseline

A reduction in size was observed on imaging studies. MRI performed 12 weeks after the initiation of sirolimus showed a maximum diameter reduction to 331.0 mm (− 25.0 mm) and volume reduction to 5098.0 mL (− 1474.5 mL) (Fig. 3a–c). The patient reported improved abdominal pressure symptoms. At 30 weeks, the maximum diameter further decreased to 310.4 mm (− 45.6 mm), with the volume reduced to 4322.7 mL (− 2239.8 mL), representing a 34.1% reduction from baseline (Fig. 3d–f). The oral mucositis did not worsen and remained manageable with topical treatment. The patient’s sense of abdominal fullness improved further, and she expressed high satisfaction with the treatment efficacy.

## Discussion

HHs are benign tumors that tend to enlarge. Therefore, follow-up imaging every six or 12 months is recommended, and no cases of malignant transformation have been reported [28, 29]. Treatment is considered in cases complicated by Kasabach–Merritt syndrome, those presenting with symptoms, such as abdominal pain, those showing a growth tendency, or those in which a definitive diagnosis cannot be established [13–16]. In such situations, surgical treatment may be considered. However, there is no consensus on the surgical approach, with hepatic resection or enucleation being the typical options [16, 30, 31]. Several studies have reported an increased risk associated with hemangiomas > 100 mm in diameter [32, 33]. Transcatheter arterial embolization is a potentially effective treatment in such cases. Most reports describe transcatheter arterial chemoembolization, in which lipiodol is combined with chemotherapeutic agents [32, 34–36]. However, as hemangiomas are benign tumors, the use of anticancer agents should be carefully considered, particularly because this approach remains off-label and is not covered by insurance. In this case, transcatheter arterial chemoembolization was considered in 2019. However, due to ethical and safety concerns associated with the use of chemotherapy for benign tumors, the procedure was not performed. Furthermore, HH in this case was too large to be effectively treated by embolization alone without chemotherapy, and selecting the target vessels for embolization was difficult thus, a therapeutic effect was not expected. Percutaneous or laparoscopic radiofrequency ablation has been proposed as a treatment option [37–41], although this technique has shown limited efficacy and a high complication rate in hemangiomas > 100 mm [42, 43]. Presently, the patient had a giant HH, measuring 356.0 mm in diameter, along with multiple hemangiomas in the surrounding normal liver parenchyma, making surgical treatment infeasible. Other treatment options pose high risks with limited therapeutic benefits. Although liver transplantation has been reported as a treatment for giant HHs, our patient did not present with circulatory or hepatic failure requiring urgent intervention. Therefore, liver transplantation is not indicated [44].

Pharmacological treatment for infantile giant HHs generally involves corticosteroids as the first-line therapy, with approximately 20–25% of patients responding to tumor regression on steroid monotherapy [45]. Agents, such as vincristine, actinomycin D, and cyclophosphamide, have been reported to be effective in steroid-refractory cases. However, the benign nature of HHs necessitates careful consideration of adverse effects and insurance coverage if using cytotoxic agents [45, 46]. Some reports have described the incidental efficacy of anti-vascular endothelial growth factor agents, such as sorafenib and bevacizumab, in adults. However, these therapies have similar limitations [47, 48]. Although propranolol is recommended as first-line therapy for infantile hemangiomas by the American Academy of Pediatrics, few studies have reported its efficacy in treating HHs [49, 50]. Here, long-term administration of propranolol was ineffective.

Sirolimus, a mTOR inhibitor with antiangiogenic properties, was approved in Japan in 2015 for the treatment of lymphangioleiomyomatosis [51]. Following the results of a large multicenter prospective phase III clinical trial, sirolimus received insurance approval in Japan in January 2024 for the treatment of intractable vascular malformations, including venous malformations [19]. Cavernous hemangiomas, the most common subtype of HHs, are classified as venous malformations according to the ISSVA classification, and exhibit features of slow-flow venous malformations [21, 52]. Such lesions are frequently characterized by abnormal angiogenesis via hyperactivation of the phosphoinositide 3-kinase (PI3K)/protein kinase B/mTOR signaling cascade [53]. Moreover, somatic mutations in the tyrosine kinase with immunoglobulin and EGF homology domains 2 (TIE2) gene, which promote PI3K activation, have been observed in > 50% of patients with venous malformations, whereas approximately 20% harbor phosphatidylinositol-4,5-bisphosphate 3-kinase catalytic subunit alpha (PIK3CA) mutations [54]. In clinical trials of sirolimus for vascular malformations, long-term efficacy was reported in 84% and 83% of patients with TIE2 and PIK3CA mutations, respectively [19]. These findings suggest that the identification of genetic mutations before treatment may help guide more effective sirolimus therapy. However, genetic testing was not performed in this case, because performing a biopsy on HH generally carries a high risk of bleeding. Additionally, her abdominal symptoms had become urgent, and she wished to begin treatment as soon as possible. Further research into genetic mutations in HH and their association with treatment response to sirolimus is required. There have been several reports on the use of sirolimus in combination therapy for infantile giant HHs [22–26]. However, in adults, only one case has been reported in which sirolimus was used in combination with glucocorticoids to treat giant HHs complicated by Kasabach–Merritt syndrome [55]. Stomatitis is a commonly reported adverse effect of sirolimus and was observed in this case; however, it was managed with a topical corticosteroid ointment [19]. Transient cytopenia occurred but was resolved promptly following dose reduction. In a large-scale multicenter prospective phase III clinical trial, the efficacy and safety of sirolimus were evaluated over a 2 year treatment period. Therefore, treatment is planned to be discontinued after 2 years. If lesion regrowth is observed thereafter, resumption of treatment will be considered, with careful monitoring for adverse effects [19].

To our knowledge, this is the first reported case of an adult with a giant HH treated with sirolimus monotherapy. After 30 weeks, a 34.1% reduction in tumor size was observed, with only mild, manageable adverse effects. Thus, sirolimus represents a promising new therapeutic option for the treatment of giant HHs in adults.

## Abbreviations

AKT: Protein kinase B
CT: Computed tomography
DIC: Disseminated intravascular coagulation
FBG: Fibrinogen
FDP: Fibrinogen/fibrin degradation products
HH: Hepatic hemangioma
ISSVA: International society for the study of vascular anomalies
MRI: Magnetic resonance imaging
mTOR: Mammalian target of rapamycin
PI3K: Phosphoinositide 3-kinase
PT-INR: Prothrombin time-international normalized ratio
TIE2: Tyrosine kinase with immunoglobulin and EGF homology domains 2
VEGF: Vascular endothelial growth factor
